# Assessment of indoor radon concentration and time-series analysis of gamma dose rate in three thermal spas from Portugal

**DOI:** 10.1007/s10661-022-10157-x

**Published:** 2022-07-26

**Authors:** Ana Sofia Silva, Maria de Lurdes Dinis

**Affiliations:** CERENA/FEUP - Centre for Natural Resources and the Environment, FEUP - Faculty of Engineering, University of Porto, Rua Dr. Roberto Frias, 4200-465 Porto, Portugal

**Keywords:** Radon, Gamma dose, Effective dose, Occupational exposure, Time-series analysis, Model

## Abstract

This work is a follow-up study on the exposure to indoor radon levels in Portuguese thermal spas. The previous research involved 16 thermal spas, where radon measurements in air and thermal mineral water were performed twice a year, from 2012 to 2016. These studies revealed concerning radon concentrations both in air and water. Therefore, a follow-up study on long-term radon measurements was conducted to estimate the year-round average radon exposure. The closer the long-term measurement is to 365 days, the more representative it will be of annual average radon levels. Continuous measurements over 1 year for the indoor radon levels are now presented for three of the 16 previously studied thermal spas, together with a time-series analysis of the gamma dose rates registered within the facilities of these thermal spas (TS). An attempt to identify possible patterns in the variation of gamma dose rates was made. Hourly gamma dose rates were modelled and forecasted using the Box–Jenkins seasonal time series models (SARIMA). The results showed that between December 2018 and November 2019, the indoor radon concentration varied from 202 to 1941 Bq/m^3^ (TS1), from 52 to 191 Bq/m^3^ (TS2), and from 937 to 1750 Bq/m^3^ (TS3). Approximately 60% of the obtained values for radon concentration in the indoor air exceed the reference level of 300 Bq/m^3^. Gamma dose rates were continuously measured with GAMMA SCOUT® detectors for hourly readings (µSv/h) between 83 and 229 days. On average, the results are similar in all considered locations and range between 0.169 and 0.264 µSv/h, although variations are different in winter and summer. The calculated effective doses ranged between 3.49 and 18.65 mSv/year (TS1), between 1.37 and 2.53 mSv/year (TS2), and between 13.89 and 22.97 mSv/year (TS3). For occupational exposure purposes, workers would be classified as category A in nine locations (out of 20), as the exposure is liable to exceed an effective dose of 6 mSv/year. For the time-series analysis, the obtained models captured the dynamics of the time series data and produced short-term forecasts. Their accuracies have been quantified by minimizing the root mean square error, the mean absolute error due to the actual forecast, and the mean absolute scaled error. The current results corroborate the conclusions of previous research and give continuous data on occupational exposure to radon for three Portuguese thermal spas. For TS1 and TS3, the indoor radon levels are much higher than the reference level. Under this circumstance, mitigation measures must be implemented to reduce the radon levels accordingly with the Euratom Directive 2013/59 and the Decree-Law No. 108/2018. In general, the gamma dose rates were below 1 μSv/h and, therefore, the contribution to the annual effective dose is not significant. Nevertheless, the variation of the gamma dose rates showed a coherent behavior with the radon progeny build up in closed spaces, as when the considered facilities were closed for certain periods. The time series analysis made it possible to fit some models to the gamma dose rate variation, and although the produced models cannot forecast exact gamma dose rates, they can provide valuable information to build sound planning and decision-making strategies in occupational exposure.

## Introduction

Radon is a radioactive gas formed by the decay series of ^238^U, which disintegrates into a series of short-lived radioactive decay products: ^218^Po, ^214^Bi, ^214^Pb, and ^214^Po. These four nuclides are solid particles with shorter half-lives than radon (3.8 days) and can attach themselves to airborne particles that may stick to the surface of bronchial tissues. Radon progeny can also form clusters and remains unattached; however, these unattached clusters can also become trapped in the lungs.

Epidemiological studies have clearly shown that long-term exposure to high radon concentrations in indoor air increases the risk of lung cancer (WHO, 2009). Radon exposure is recognized as the second leading cause of lung cancer after tobacco and one of the leading causes of lung cancer in non-smokers (Alali et al., 2019; Alghamdi et al., 2019; Allab, 2019; Berens et al., 2017; Robertson et al., 2013; Vukotic et al., 2019).

For most people, radon and progenies are the most significant source of exposure to natural radiation. According to UNSCEAR (2017), the overall average individual exposure dose to all sources of radiation in the environment is 3.0 mSv. Approximately 80% comes from exposure to natural radiation, and about 40% is attributed to radon exposure (1.2 mSv).

High indoor radon concentrations are often associated with particular geological formations, such as soils rich in granite substrates with uranium and thorium mineralizations. When uranium decays in soils and rocks, radon is released through the porous spaces by molecular diffusion or convection into the air or dissolving into groundwater. The entry of radon gas into buildings occurs through cracks in floors, construction joints, walls, and pipes, which becomes a potential health hazard when it accumulates in enclosed spaces such as basements or lower-level structures (Pugliese et al., 2014).

When present in surface waters, such as lakes and rivers, radon is released by agitation as it passes over rocks and soils and dissipates into the outdoor air. Groundwater from wells and boreholes usually contains higher radon concentrations than surface waters. In specific circumstances, very high radon concentrations are found in drinking-water supplies from these sources. Therefore, some of the dissolved radon may be released into the indoor air through water usage, which will be of concern for inhalation and the indoor radon from other sources. On the other hand, its contribution is not constant since radon is only released when there is agitation of the water, such as when a tap or shower is turned on. This is supported by UNSCEAR’s (2000) studies which concluded that, on average, 90% of the dose attributable to radon in drinking water comes from inhalation rather than ingestion.

Many important factors strongly affect indoor radon levels, such as indoor air conditions, ventilation systems, and meteorological factors, modifying diurnal and longer-term indoor variations. Indoor radon levels can vary significantly over time. It is common to see radon levels change by a factor of two to three over one day. Variations from season to season can be even larger; higher radon levels are usually observed during winter months when houses are sealed up. In particular, for workplaces, the heating and air-conditioning systems have significantly influenced the radon concentration in indoor air (Marley, 1999; Marley et al., 2000; Nivolov et al., 2012; Panatto et al., 2006). Some studies showed that the operation of water-heated central heating systems in above-ground workplaces could decrease radon and radon progeny levels by more than 40% during the heating period of a typical working day (Marley et al., 2000). Therefore, long-term measurements are needed to give a more accurate indication of the annual average radon concentration. Long-term measurements are three to 12 months in duration.

In what concerns dose assessment, several studies are devoted to determining doses from alpha particles emitted by radon and its progeny. But the contribution of gamma radiation from radon progeny to total dose has mostly been neglected so far.

There has been an increasing concern about the considerable high concentration of radon and progeny observed in thermal spas where mineral water resources are used in natural treatments and health therapies. This exposure represents an additional radiation burden to patients and, particularly, workers, who may be exposed for long-term periods over many years at their workplace.

The EU has identified spa therapy as one of the professional activities with potential for radon exposure in the directive 96/29/EURATOM. The most recent Directive 2013/59/EURATOM has, for the first time, set down a framework for controlling the exposure to natural radiation sources arising from work activities, including thermal spas, aiming to assure better protection in workplaces and dwellings as well. The document introduces radon gas into the radiological protection system and establishes 300 Bq/m^3^ for the indoor radon concentration as a reference level. In addition to this, the occupational exposure regulation for radon in workplaces has a categorization of exposed workers in which for a dose above 6 mSv/year, the situation should be managed as a planned exposure situation. Below this value, the requirement is to keep the exposure under review. With the transposition of the 2013/59/EURATOM directive (December 2018 in Portugal), several requirements are mandatory for radon measurements and assessing the external gamma dose in specific workplaces.

This work is a follow-up study on indoor radon exposure in Portuguese thermal spas. The previous research involved 16 thermal spas where radon measurements were performed in indoor air and thermal mineral water twice a year between 2012 and 2016 (Silva et al., 2013, 2014, 2016, 2020; Silva & Dinis, 2015a, b, 2016, 2017a, b, 2018a, b, c). The previous studies evaluate indoor radon concentration in workplaces and dwellings, addressing the contribution of radon exposure from a non-occupational environment to the total effective dose. Indoor radon was measured twice a year for periods between 25 and 45 days, and radon in water was measured only once in several treatment facilities. The results from these studies revealed concerning radon concentrations both in air and water, reaching in some cases 4300 Bq/m^3^ in air and 3600 Bq/L in water. Indoor radon levels were equally high in the worker’s dwelling.

Within the scope of this work, a long-term study of the indoor radon activity concentration and indoor gamma dose rate, measured simultaneously for 12 months, was conducted to evaluate the radon concentration and the time-series analysis of gamma dose rates registered in three Portuguese thermal spas between December 2018 and November 2019. The aim was to assess the radiation burden on working personnel through as much as possible continuous measurements and identify potential patterns in the variation of gamma dose rates through the time series analysis. This analysis relies on the fact that registers taken over time may have an internal structure (such as autocorrelation, trend, cycle, or seasonal variations) that should be accounted for. The experience gained from the previous works and the results were considered in the present study.

## Materials and methods

### Study area

**Fig. 1:**
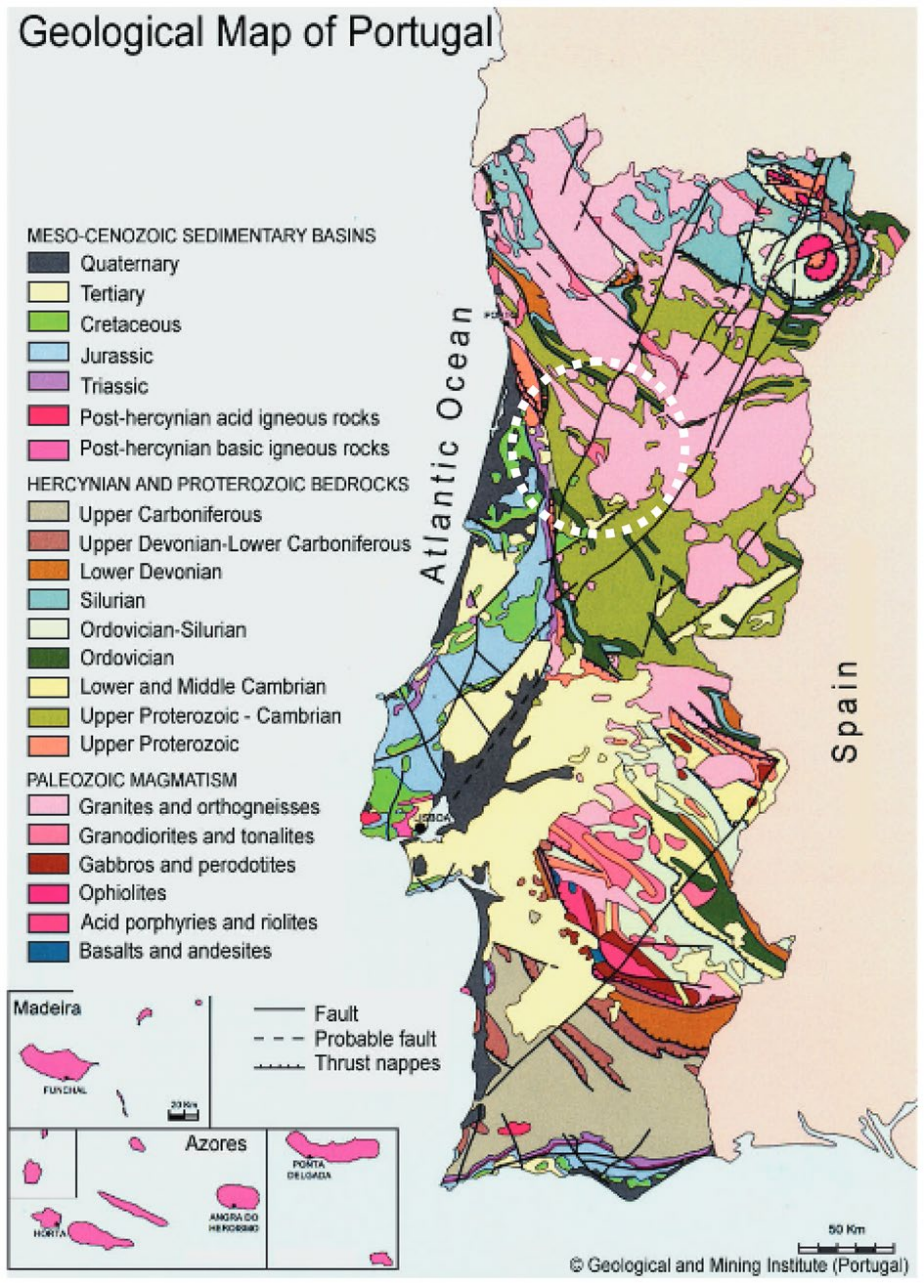
Geological map of Portugal

Most of the Portuguese thermal spas are located in the north and centre regions, where it is possible to find geological formations with different radiological profiles according to their potential to generate radon: low-radon production or high-radon production (Fig. 1). For the present study, the managers of three thermal spas located in the centre region of Portugal, identified as TS1, TS2, and TS3, allowed free access to their facilities to perform measurements for radon levels and gamma dose rates.

In terms of geological settings, TS1 is located in a region of metasedimentary rocks prevailing outcrops of red and grey sandstones from the Carbonic and phyllite shales from the Neoproterozoic; TS2 is located in a region with granite and schist substrate where it is possible to identify outcrops of two-mica hercinic granites and shales, metagrauvaques and metaquartzites from the Schist-Greywacke Complex, and TS3 is located in a region mainly with a granitic substrate (granites and ortogneisses). These facilities represent 8% of the total existing thermal spas (35) in Portugal.

The thermal spa facilities under study provide several medical treatments, comprising treatment rooms, thermal water infrastructure, and water emerging (either naturally or piped from the spring and across which the water is channelled from the spring to the baths or other installations such as pools and showers), technical areas, and administrative service rooms.

All treatment rooms have mechanical ventilation systems (pools, showers, and inhalation therapy rooms) and centralized heating systems. In many cases, natural ventilation is primarily used (except in swimming pool areas) by opening the windows whenever it is possible, and a mechanical system is used for forced ventilation.

### Radon measurements

Data were continuously collected between December 2018 and November 2019 for indoor radon concentrations (*C*_Rn_) and gamma dose rates (GDR). A survey for total gamma radiation (TGR) was performed at the beginning of the study at each facility of the three thermal spas. It should be noted that for TS2, the facilities include two buildings, identified as TS2-E1 and TS2-E2 in this study.

The concentration of indoor radon was measured in several different locations of each thermal spa, namely Bartholet, collective pool, hydromassage cabins, nozzle showers, ORLs (inhalation therapy rooms), reception halls, spa pools, technical areas, thermal pools, thermal spa halls, treatment hall, and Vichy shower, using CR-39 solid-state nuclear track detectors (SSNTD) calibrated and supplied by a certified laboratory (Natural Radioactivity Laboratory of the Earth Sciences Department, in the University of Coimbra, Portugal).

The detectors were affixed at each location at a height between 1 and 2 m from the ground for periods between 94 and 113 days. After each exposure period, the detectors were replaced by an equivalent exposure period. The recovered detectors were sent to the Natural Radioactivity Laboratory where they were etched in 25% NaOH solution at 90 °C for 270 min. An automatic microscope reader counted the number of tracks in an area of 1 cm^2^ on each film. The background track density was then subtracted and related to radon concentration level using a calibration factor obtained by exposing detectors of the same batch in a certified calibration chamber. This laboratory regularly participates in comparison exercises with other laboratories to estimate statistical uncertainty (analytical error of less than 10% of the obtained value). The detection limit using the procedure described is 5 Bq/m^3^.

### Gamma radiation assessment

Ambient gamma dose rates were measured in thermal pools and ORLs of thermal spas with GAMMA SCOUT® (GS3) detectors. The GS3 calibrated instruments can measure gamma radiation (*γ*), alpha and beta radiation (*α* + *β*), and alpha and beta and gamma radiation (*α* + *β* + *γ*). These instruments are calibrated and inspected at the factory every 2 years of use.

For this study, the device was set to measure gamma dose rates, with hourly readings (µSv/h) and for a period between 83 and 229 days. These measurements coincided with the measurements of the indoor radon concentrations. Descriptive statistics were applied to characterize the gamma dose rate registers.

A portable Saphymo-SRAT SPP2 NF scintillometer was used for the measurements of total gamma radiation. This detector is a NaI(Tl)—sodium iodide activated with thallium—scintillation crystal, and the operation range for gamma radiation is from 0.02 to 30 μSv/h. The unit of measurement used by the SPP2 is cps (counts per second). This equipment is calibrated and verified at factory every year.

The measurements of total gamma radiation were taken during the first visit to the facilities of each thermal spa with a focus on the therapy inhalation rooms (ORLs) and thermal pools.

### Effective dose assessment

The internal dose due to radon inhalation (ID_Rn_, mSv/year) was determined using the following equation (ICRP, 2018):

1 $${ID}_{Rn} = {C}_{Rn} \times {D}_{CF} \times {T}$$

where ID_Rn_ is the inhalation dose due to indoor radon concentration (mSv/year), *C*_Rn_ is the average indoor radon concentration (Bq/m^3^), *D*_CF_ is the dose coefficient conversion factor for radon, and *T* is the exposure period, i.e. the number of working hours in a year (2000 h/year). The dose coefficient depends on the equilibrium factor, *F*, between radon gas and its progeny. By adopting the standard assumption of *F* = 0.4 for most indoor situations, the dose coefficient corresponds to 6.7 × 10^−6^ mSv per Bq h m^−3^ (ICRP, 2018). Using this dose coefficient, the exposure to radon at the upper value of the national reference level of 300 Bq m^−3^ corresponds to an annual dose of 4 mSv at work and 14 mSv at home (ICRP, 2018).

The external dose (ED) was obtained by multiplying the average of the measured gamma dose rates (GDR, mSv/h) by the exposure period of 2000 h/year (Dinis & Silva, 2018; IAEA, 2014; ICRP, 2018; UNSCEAR, 2000):

2 $${ED} = {GDR}\; \times {T}$$

The annual effective dose results from the sum of the internal dose with the external dose.

### Autocorrelation function

A time series is a sequence of measurements of the same variable(s) made over time. Usually, the measurements are made at evenly spaced times. The autocorrelation measures the linear relationship between lagged values of a time series. The plot of the autocorrelation of a time series by lag is called the autocorrelation function (ACF), also known as correlogram (plot of the autocorrelation versus time lag) or an autocorrelation plot. The plots graphically represent the strength of a relationship between an observation at time *t* and the observations at previous times.

From December 2018 to November 2019, gamma dose rates were continuously registered at least one location from each thermal spa. At each location, the dose rates were continuously measured at each hour for periods between 83 and 165 days in TS1 and during 229 days in TS2 (E1). Hourly sampled time gamma dose rates were obtained for the following periods:

- From late December to mid-March and from late August to late November (83 and 95 days, respectively) in TS1 (thermal pool).
- From late December to late August (165 days) in TS1 (ORL).
- From mid-April to late November (229 days) in TS2 (ORL).

In the thermal pool (TP) from TS1, no observations were registered during two of the four measurement periods: 14/03/2019–15/04/2019 and 15/04/2019–26/08/2019 due to a failure in the GS3 equipment. Data on dose rates collected from TS3 had to be rejected due to equipment failure during the measurement period.

Autocorrelation plots were obtained for the considered time series trying to identify patterns such as tendencies for observations to vary periodically, either as seasonality, periodicity, or cyclic pattern in time series. Therefore, possible trends of periodicities variations were explored for gamma dose rates and it was possible to adjust a mathematical model, in some cases, by the ARIMA modelling exploratory procedure. An attempt of forecasting the gamma dose rates was made for a short-term prediction beyond the actual data. The details of the theoretical support of time series analysis with ARIMA modelling can be found in the specialized literature (Box & Jenkins, 1970; Box & Tiao, 1975; Box et al., 2008).

The time series analysis was performed with the IBM®SPSS® Statistics 28.0.0.0 software. The procedure is described in the next section.

## Results and discussion

### Indoor radon concentration

The results of the indoor radon measurements are presented in Table 1, according to the location and the exposure time of the CR-39 detectors.

**Table 1:** Radon concentrations measured in the indoor air of the studied thermal spas (Bq/m.^3^)

Location	TS1	TS1	TS2 E1	TS2 E2	TS3	TS1	TS2 E1	TS2 E2	TS3
	21/12/2018–15/04/2019	15/04/2019–26/08/2019				26/08/2019–28/11/2019			
Bertholet	1747	1133	-	-	-	958	-	-	-
Collective pool	-	-	52	-	-	-	-	-	-
Hydromassage cabin	415	366	-	-	-	295	-	-	-
Nozzle shower	1649	1310	-	-	992	1147	-	-	1212
ORL	1283	1941	89	92	937	1083	126	114	1146
Reception	313	280	-	-	-	202	-	-	-
Spa pool	437	383	-	-	-	333	-	-	-
Technical area	-	-	-	-	1130	-	-	-	1474
Thermal pool	344	305	-	131	1679	286	67	156	1750
Thermal spa hall	-	-	-	187	-	-	-	191	-
Treatment hall	-	-	-	149	-	-	-	187	-
Vichy shower	801	1175	161	144	1400	491	187	171	961
Number of measurements	8	21				21			
Range of indoor radon	313–1747	52–1941				67–1750			
Standard deviation	601	603				530			

“-” The location does not exist or was not assessed in this TS

The results show that, between December 2018 and November 2019 and for TS1, the indoor radon concentration varied from 202 to 1941 Bq/m^3^, with an arithmetic mean of 778 ± 527 Bq/m^3^ and a geometric mean of 612 ± 527 Bq/m^3^. For TS2, radon concentration ranged from 52 to 191 Bq/m^3^ and for TS3, from 937 to 1750 Bq/m^3^ (Table 1).

Approximately 60% (30/50) of the radon concentration values in the indoor air exceed the reference level of 300 Bq/m^3^.

Higher values were registered in TS1, 1747 Bq/m^3^ and 1941 Bq/m^3^, during winter and summer, respectively. Usually, higher radon concentrations are expected to occur during winter. Even the lowest value of indoor radon level during winter (313 Bq/m^3^) is above the reference level. The high values registered in TS1 (1747 Bq/m^3^, 1649 Bq/m^3^, and 1283 Bq/m^3^) may be due to the lack of optimization of the existing ventilation system, in particular, in ORL, but also in Bartholet and showers rooms. In Portugal, most of the granitic regions are situated in regions where winters are colder than in the rest of Portugal, and therefore, in the winter, windows often stay closed, not allowing proper ventilation into the buildings. So, the origin of the high values found in TS1 could be explained by the geological composition of the soils, enhanced by the poor ventilation of the building. Nevertheless, and although the radon levels decrease throughout the spring, summer, and autumn periods, with the exception of the reception of TS1, radon levels stay close to 300 Bq/m^3^ or are much higher.

In what concerns the TS2, and for both buildings (E1 and E2), all measured values for indoor radon concentration are below 300 Bq/m^3^, with an arithmetic mean of 138 ± 43 Bq/m^3^ and a geometric average of 129 ± 43 Bq/m^3^. The highest value (191 Bq/m^3^) was registered between August and November 2019 in the thermal spa hall of E2. For this facility, regarding its location and the geological setting of the region, higher indoor radon levels could be expected. In Portugal, the highest radon concentrations were observed in districts whose geological substrate is mainly composed of granites (Guarda and Viseu), which are effectively the lithic variety that typically incorporates the highest uranium contents. Moreover, uranium mineralization phenomena are also frequent in these regions where uranium was exploited at several locations from 1913 to 2001 (Domingos et al., 2020).

The radon levels were assessed at workers’ dwellings from this facility (TS2-E2) during 42 days in a previous study carried out in 2014 (Silva & Dinis, 2016). On average, the values were 250 Bq/m^3^, higher than those registered at the respective workers’ workplace. Therefore, the ventilation system in place (natural and artificial) at the workplaces seems to lower the indoor radon levels.

For TS3, all measured values are above the reference level (300 Bq/m^3^). The minimum value was 937 Bq/m^3^, and the maximum was 1750 Bq/m^3^, with an arithmetic mean of 1268 ± 279 Bq/m^3^ and a geometric mean of 1239 ± 279 Bq/m^3^, between April and November 2019. These high radon levels, registered in the indoor air of TS3, are primarily a result of the geology of the region where this thermal spa is located, mainly composed of granitic substrate, and where the ventilation system (only natural ventilation) is not sufficient to decrease the radon levels. Many other factors affect the indoor radon levels, such as construction properties and characteristics, and soil permeability but are not the focus of this work (Xie et al., 2015). Previous studies conducted in 2014 concerning the assessment of radon levels at workers’ dwellings from this facility also presented values above the reference level (on average 600 Bq/m^3^ for an exposure period of 43 days) (Silva & Dinis, 2016).

### Total gamma radiation and gamma dose rates

Table 2 presents the results for the measurements of total gamma radiation.

**Table 2:** Total gamma radiation (cps)

	Location	TS1	TS2 (E1)	TS (E2)	TS3
Ground floor	Thermal pool	100	-	250	250
	Collective pool	-	150	-	-
	Thermal pool hall	100	-	250	-
	Treatment hall	-	-	250	-
	ORL	100	-	250	-
	Vichy shower	100	200	250	1000
	Bartholet	100	-	-	-
	Nozzle shower	100	-	-	-
	Reception	100	-	-	-
	Double cabin	100	-	-	-
	Spa pool	100	-	-	-
	Technical area	-	-	-	250
1° floor	Emanatory	100	-	-	-
	ORL	-	-	-	200
	Nozzle shower	-	-	-	250
2° floor	ORL	-	200	-	-

“-” The location does not exist or was not assessed in this TS

There is no significant variation for the total gamma radiation; the results are low and similar (100–250 cps). However, an abnormal situation was registered at the ground level of the Vichy shower in TS3 from the water collection system (floor trench drain), with 1000 cps. This is probably caused by the water accumulated in the trench drain.

Gamma radiation dose rates were measured in the ORL rooms of TS1 and TS2 (E1) for a period ranging between 83 and 229 days. The results are presented in Table 3. In what concerns TS2 (E2) and TS3 measurements, the results were rejected due to an equipment failure.

**Table 3:** Gamma dose rates (µSv/h)

TS	Location	Exposure period	No. days	Readings	Average	Min	Max
TS1	Thermal pool	21–12-2018/14–03-2019	83	1843	0.237	0.184	0.830
		26–08-2019/28–11-2019	95	2253	0.169	0.151	0.193
	ORL	21–12-2018/14–03-2019	165	1993	0.191	0.161	0.245
		14–03-2019/15–04-2019		768	0.198	0.170	0.234
		15–04-2019/26–08-2019		3191	0.207	0.171	0.257
TS2	ORL	15–04-2019/26–08-2019	229	3189	0.264	0.235	0.783
		26–08-2019/28–11-2019		2254	0.262	0.234	3.366

On average, the results are similar in all considered locations. Nevertheless, maximum values in the thermal pool from TS1 are very different in winter (higher values) and summer (lower values). In particular, there was a sequential increase in the gamma dose rates from March 1, 2019 (Friday) to March 6, 2019 (Wednesday) of 0.285 µSv/h to 0.830 µSv/h, respectively. In comparison, indoor radon levels generally tend to be higher in winter because the windows and doors are usually closed, resulting in increased radon accumulation in closed premises.

Also, the registered maximum values in the ORL from TS2 are very different when comparing the two measurement periods. However, the 3.366 µSv/h dose rate recorded in TS2 is an isolated value probably caused by an overflow of the equipment (Duarte, 2013). Despite all the protective capacitors on the circuit board, strong high-frequency fields can cause ionization that does not originate from *α*, *β*, or *γ* radiation. The two most common causes known are a cell phone within a few centimetres of the GS3, which develops a relatively strong field when transmitting, or certain fluorescent lamps, which create such a field on lighting up. Removing the cell phone from the direct vicinity of the equipment eliminates the interference. Therefore, the high value of 3.366 µSv/h is not considered in the data set gamma dose rates.

### Estimation of the effective dose

The effective dose was estimated for TS1 (thermal pool and ORL) and TS2 (E1- ORL), according to Eqs. (1) and (2), considering the average values of radon concentration and gamma dose rates for each location. The results are presented in Table 4 where, for TS3, the results refer only to the contribution from the internal exposure (internal dose). Nevertheless, the most significant contribution to the effective dose is due to the radon concentration.

**Table 4:** Estimation of internal dose, external dose, and effective dose (mSv/year)

Thermal Spa	Location	Internal dose (mSv/year)	External dose (mSv/year)	Effective dose (mSv/year)
TS1	Bartholet	16.59	-	16.59
	Thermal pool	4.16	0.406	4.57
	Hydromassage cabin	4.76	-	4.76
	Nozzle shower	18.13	-	18.13
	ORL	18.65	0.397	19.05
	Reception hall	3.49	-	3.49
	Spa pool	5.12	-	5.12
	Vichy shower	10.36	-	10.36
TS2 (E1)	ORL	1.42	0.526	1.95
	Vichy shower	2.33	-	2.33
TS2 (E2)	ORL	1.37	-	1.37
	Treatment hall	2.24	-	2.24
	Thermal pool	1.92	-	1.92
	Thermal spa hall	2.53	-	2.53
	Vichy shower	2.10	-	2.10
TS3	Nozzle shower	14.69	-	14.69
	ORL	13.89	-	13.89
	Technical area	17.29	-	17.29
	Thermal pool	22.97	-	22.97
	Vichy shower	15.54	-	15.54

“-” The location does not exist or was not assessed in this TS

According to the Decree-Law No. 108/2018, the annual effective dose limit for exposed workers is 20 mSv/year or 100 mSv/5 years, provided that during the 5 years, this value does not exceed an effective dose of 50 mSv/year. The effective dose, calculated for TS1 and TS2, falls within the limit of 20 mSv/year for occupational exposure.

For TS3, although referring only to the dose resulting from radon exposure, the estimated value of ~ 23 mSv/year in the thermal pool does not meet the dose limit established by the radiological protection legislation.

For monitoring and control purposes, workers from TS1 (thermal pool, hydromassage cabin, reception hall, and spa pool) and TS2 (both E1 and E2) would be classified as category B, according to the Decree-Law No. 108/2018, since the effective dose may be considered below 6 mSv/year (the contribution from the external exposure is negligible). In this case, the classification implies quarterly monitoring of the dosimetry. For all other cases, where workers’ exposure is liable to exceed an effective dose of 6 mSv per year, workers are classified as category A. Therefore, these workplaces shall be managed as a planned exposure situation where dose limits apply, being necessary to determine which operational protection requirements need to be set. It is required that category A workers be systematically monitored based on individual measurements, for example, using personal dosimeters. The individual monitoring of workers classified as category A should be performed once monthly. The measures taken towards optimization of radiation protection may include a change in the organization, method or regime of work, modification of the workplace, or modification of the ventilation system. In the Decree-Law No. 108/2018, it is planned that, for the purpose of radiation protection, arrangements are made on workplaces, including the classification of the workplaces into different areas, such as controlled areas and supervised areas.

The WHO recommends that, whenever possible, the level of radon in indoor air be kept below 100 Bq/m^3^. Using ICRP (2018) dose coefficient, the exposure to radon at the upper value of the national reference level of 300 Bq/m^3^ corresponds to an annual effective dose of 4 mSv at work and 14 mSv at home.

### Statistical distribution of gamma dose rates

**Fig. 2:**
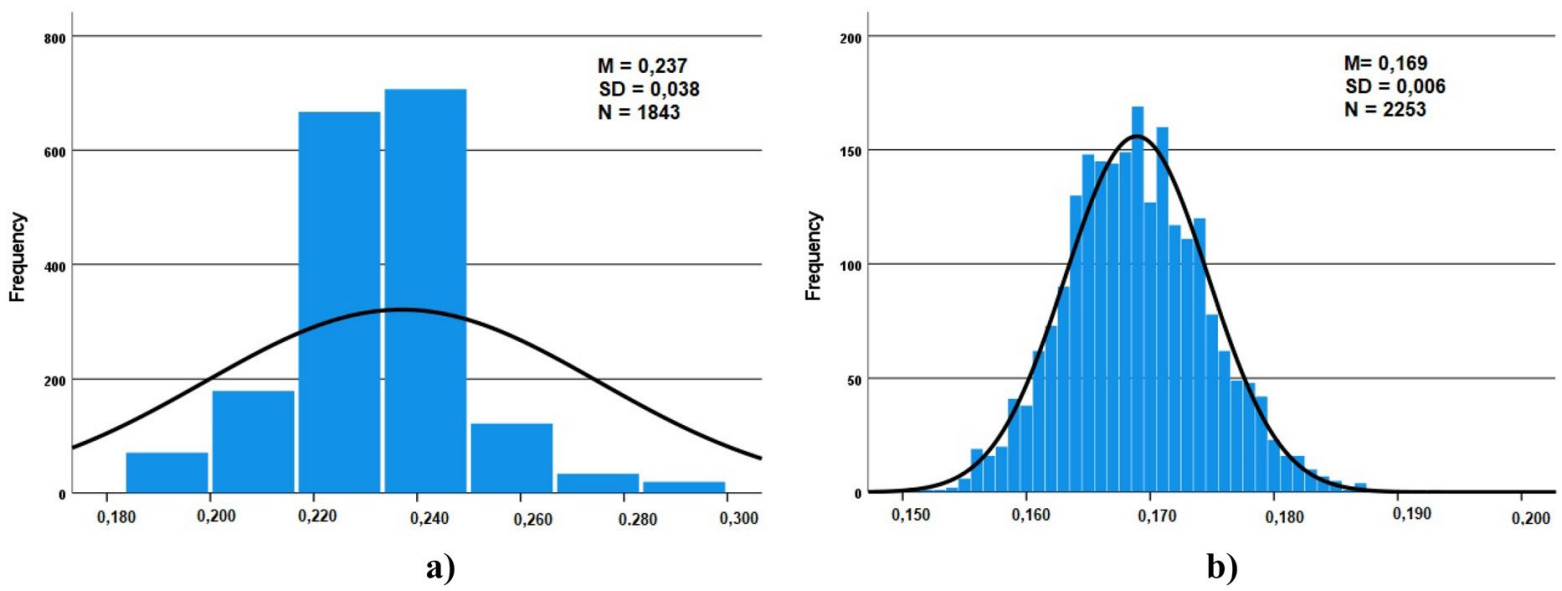
Frequency distribution of the gamma dose rates: **a** TS1-TP1 (winter) **b** TS1-TP2 (summer)

**Fig. 3:**
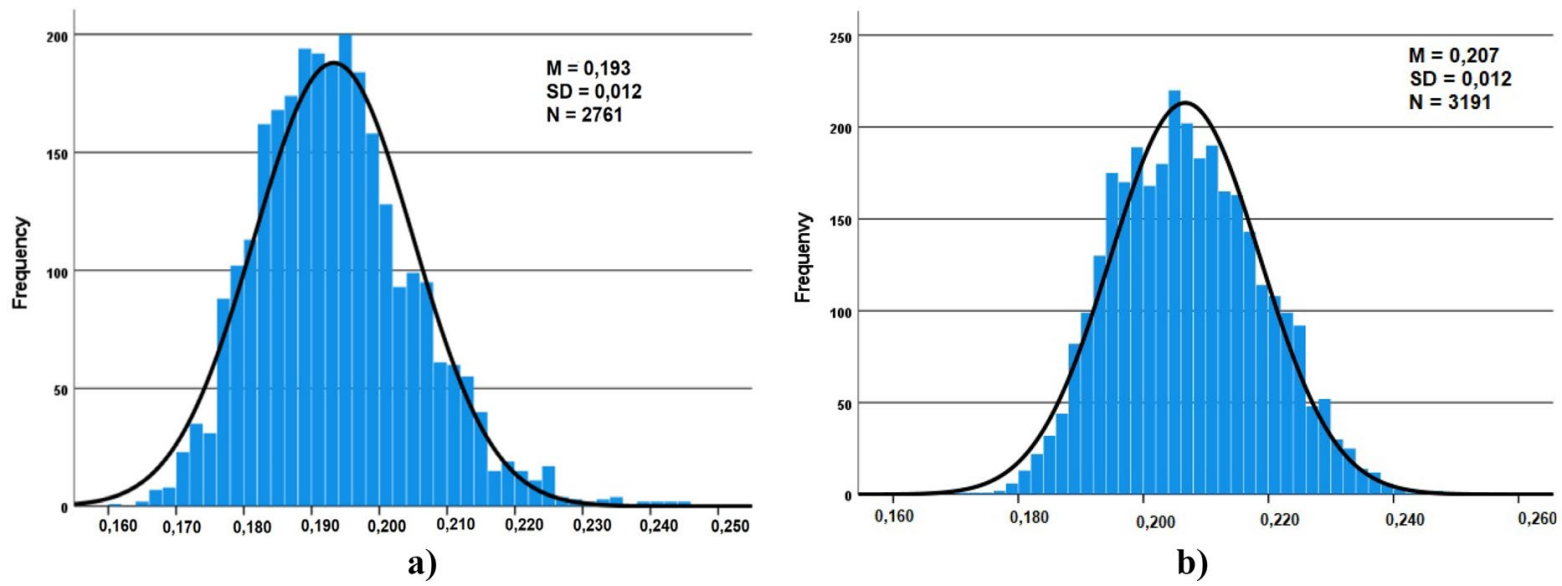
Frequency distribution of the gamma dose rates: **a** TS1-ORL1 (winter/spring) **b** TS1-ORL2 (spring/summer)

**Fig. 4:**
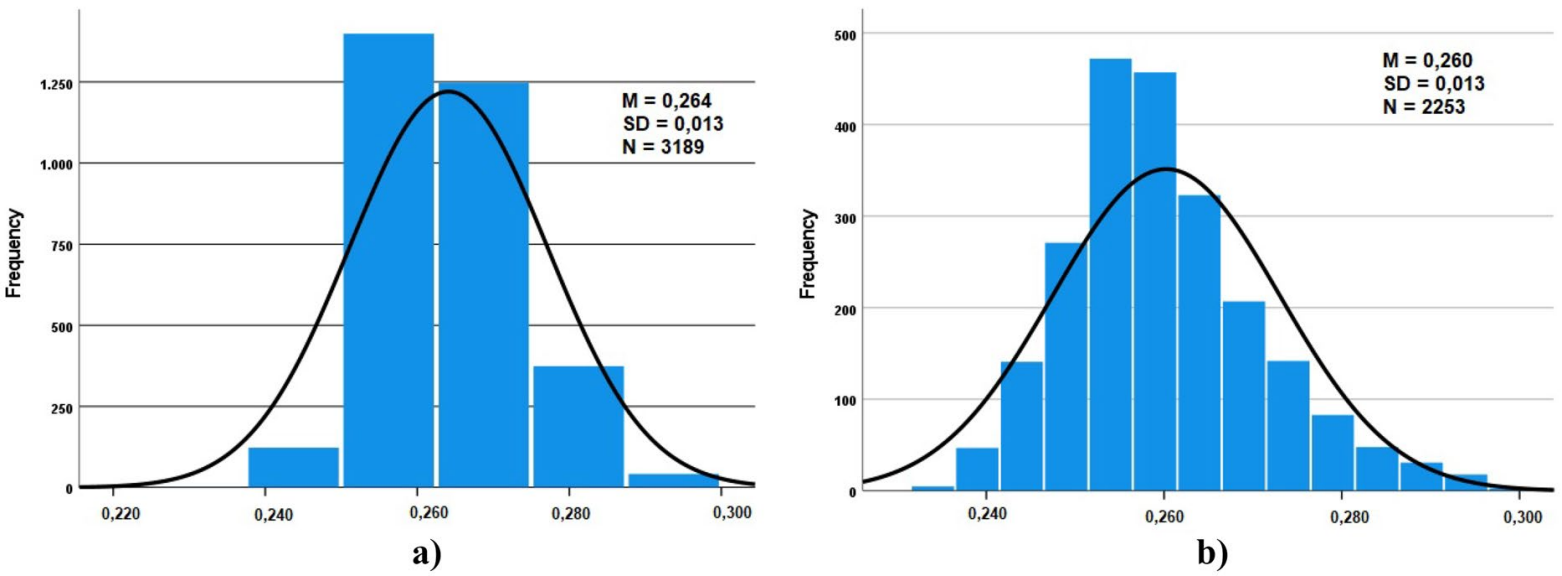
Frequency distribution of the gamma dose rates: **a** TS2-ORL1 (spring/summer) **b** TS2-ORL2 (summer/autumn)

The frequency distribution and summary statistics for the mean (*M*), standard deviation (SD), and the number of observations (*N*) of the registered gamma dose rates are presented in Figs. 2, 3, and 4.

Similar distributions were obtained for the different considered locations. The theoretical distribution that best fits the experimental distribution appears to be the normal function, the resulting mean values for each location range from 0.169 to 0.264 µSv/h.

The normal distribution tells us that a significant concentration of the registered values is around the mean value (peak) and that the data present the same proportion of high and low values (tales).

### Time series analysis

**Fig. 5:**
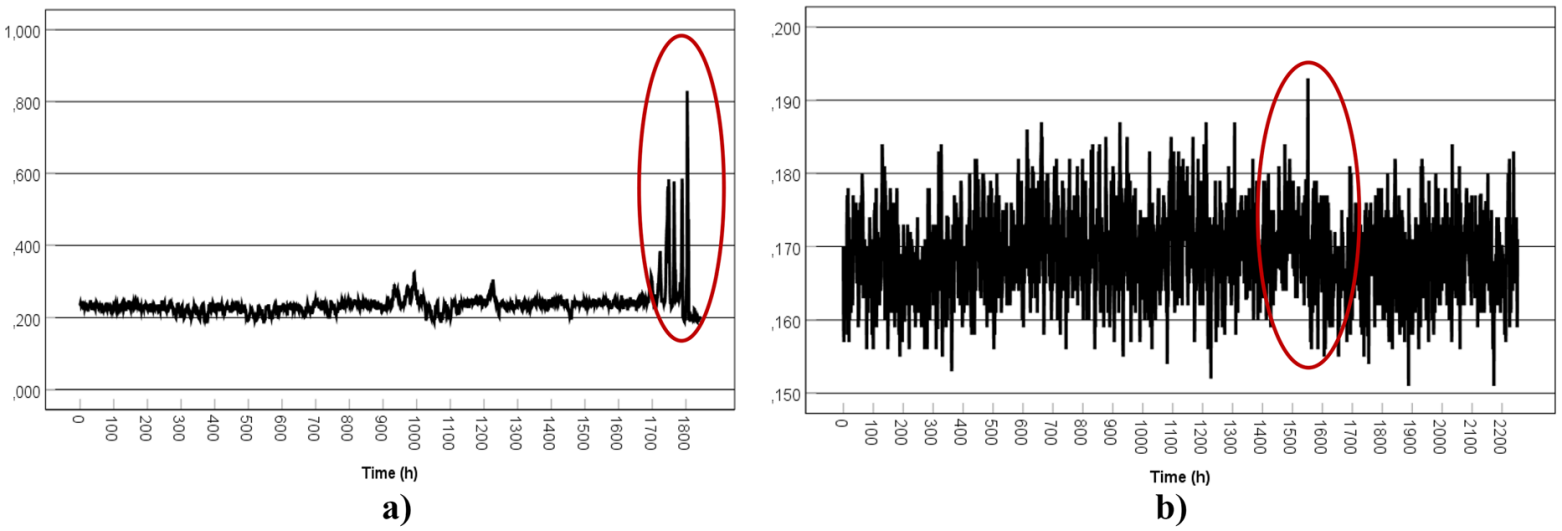
Plots of gamma dose rates per hour (µSv/h): **a** TS1-TP1; **b** TS1-TP2

For the first location, the thermal pool from TS1 was considered a data set of 1843 hourly observations of gamma dose rates (µSv/h) from 21/12/2018 (Friday) to 14/03/2019 (Thursday) (Fig. 5a). The second temporal horizon corresponds to a data set of 2253 observations from 26/08/2019 (Monday) to 28/11/2019 (Thursday) (Fig. 5b).

In Fig. 5a it is possible to see the presence of several abnormal observations at the end of the measurement period, which extended from 02/03/2019 (Saturday) to 06/03/2019 (Wednesday). This period corresponded to the Carnival festive season in 2019. The peak value (0.830 µSv/h) was registered on 06/03/2019 from 14h17 to 15h17 (Ash Wednesday).

Some remarks can be made through an initial analysis of the registers from the first period:

- The series is likely to have a seasonal component, as in the chart we can observe up and down oscillations consecutively, which indicates a cyclical movement;
- The series does not have an explicit trend;
- There are some disturbances present in the series.

For the second measurement period (TS1-TP2), the registered observations are more constant without abnormal values. The highest value (0.193 µSv/h) was recorded on 30/10/2019 (Wednesday) from 06h41 to 07h41. A few outliers (14 records out of 2253) correspond to values from 0.151 to 0.153 µSv/h and from 0.185 to 0.187 µSv/h, that were recorded from mid-September up to the end of October 2019. For this period, the higher values were registered during the night (during the closed premises period), while the lower values were observed at the end of the afternoon. For radon gas, this would be the consequence of the ventilation of the place during all day.

**Fig. 6:**
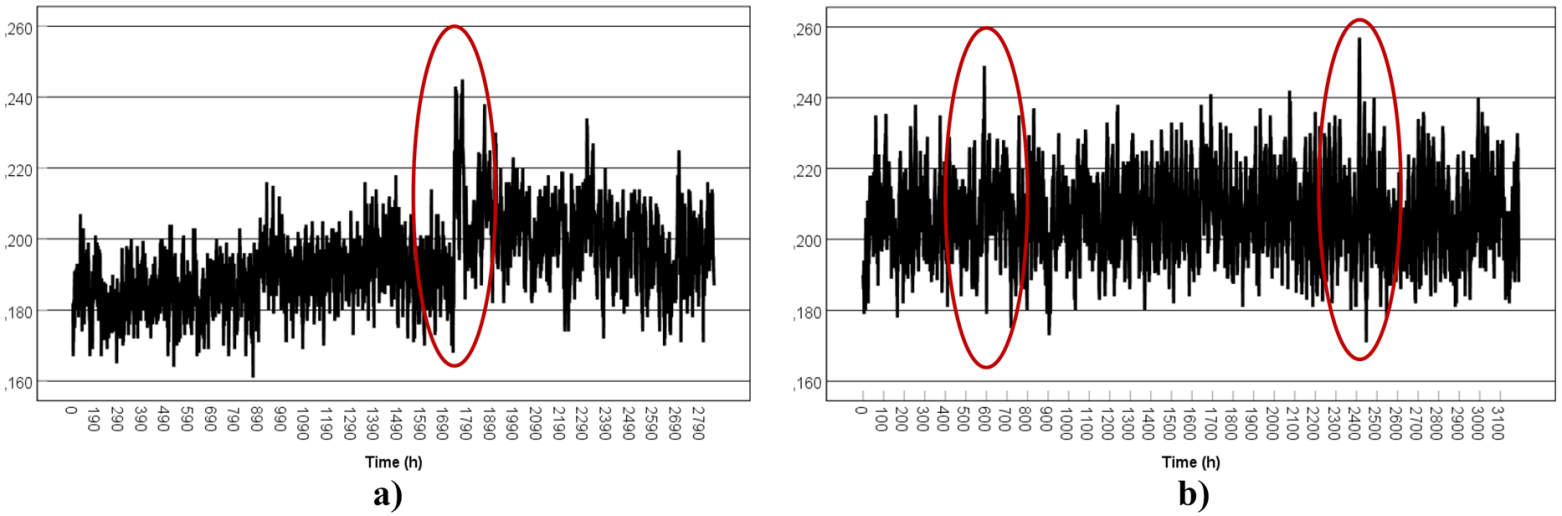
Plots of gamma dose rates per hour (µSv/h): **a** TS1-ORL1 **b** TS1-ORL2

For the second location, the ORL from TS1, it was possible to collect data in three periods in a row: 21/12/2018–14/03/2019 (1993 observations), 14/03/2019–15/04/2019 (768 observations), and 15/04/2019–26/08/2019 (3191 observations). The data from the first and the second periods were merged in one single data set for this analysis (Fig. 6a).

During the first measurement period, it is possible to observe some disturbances on the registers with an increase in the gamma dose rates. This increase corresponds to the period between 28/02/2019 (Thursday) and 12/03/2021 (Tuesday), which includes the Carnival festive season as mentioned before, meaning probably the closure of the facility. All values equal to or above 0.219 µSv/h were identified as outliers (61 registers out of 1993) and one observation of 0.161 µSv/h as well.

For the second period (Fig. 6b), the observations are more constant over time. Nine outliers were identified for values between 0.241 and 0.257 µSv/h, registered mostly on 25/07/2019 during the nighttime and at 0.171 µSv/h registered at 26/07/2009 between 13h11 and 14h11.

For both cases, the higher values were registered mostly during the nighttime, while the lowest values were registered mostly during the daytime.

**Fig. 7:**
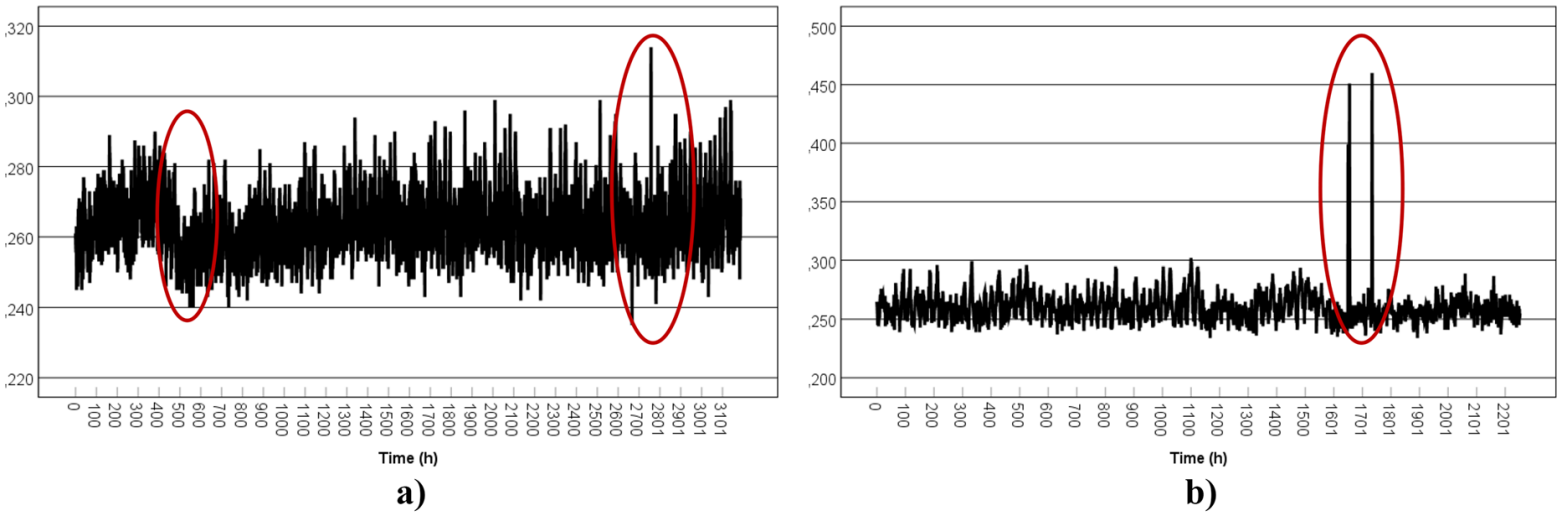
Plots of gamma dose rates per hour (µSv/h): **a** TS2-ORL1 **b** TS2-ORL2

For the third location, the ORL from TS2, 3189 observations were collected during the first period from 15/04/2019 to 26/08/2019 and 2254 observations were registered for the second period from 26/08/2019 to 28/11/2019 (Fig. 7a, b). One abnormal value was observed for the first period: 0.783 µSv/h on 08/08/2019 (Thursday) from 09h50 to 10h50. During the second period, an abnormal value of 3.366 µSv/h was observed on 31/10/2019 (Thursday) between 11h53 and 12h53. Both isolated values were removed from the respective data set for this analysis as it was caused by an overflow of the equipment, as mentioned before.

During the first measurement period, the data present oscillations consecutively. There are some disturbances, however, at the beginning and the end of this period. It was possible to identify 73 outliers (68 at the higher values and five at the lower values). A decrease of the gamma dose rate could be observed at 08/05/2019 from 06h50 to 07h50 and from 22h50 to 23h50, at 09/05/2019 from 09h50 to 10h50, at 16/05/2019 from 00h50 to 01h50, and also at 04/08/2019 from 13h50 to 14h50. The peak value (0.314 µSv/h) was registered at 08/08/2019 from 08h50 to 09h50.

Two perturbances can be observed for the second period: 03/11/2019 (Sunday)—between 05h53 and 06h53, and 06/11/2019 (Wednesday)—between 18h53 and 19h53. All observations above 0.286 µSv/h were identified as outliers (63 registers in 2254). No outliers were identified at the lower values.

The higher values were registered mainly during the nighttime for both periods, while the lowest values were registered mainly during the daytime. This pattern, verified in all facilities, coincides with the closing period and can be indirectly linked to radon build-up.

The decay of radon and thoron by alpha emission produces a short-lived progeny with half-lives ranging from 3.0 × 10^−7^ s to 10.64 h (e.g. ^214^Pb, ^214^Bi and ^212^Po, ^212^Bi). The decay of these radioisotopes through their radioactive progeny produces significant gamma and beta radiations. These decay products are particulate and can attach themselves to suspended particles in the air, remain unattached, and be deposited or implanted in surfaces, increasing the gamma-radiation dose depending on the available radionuclides, a function of radon and thoron emanation (Burnett et al., 2010; Sun et al., 2010). Several environmental factors can influence the airborne dose rate and deposited radon decay products, in particular smoking, which was considered the environmental factor that had the most significant temporal and spatial effects on airborne radon decay product dose rates (Steck et al., 2019). However, in the studied facilities, this factor does not exist as smoking is not allowed. Other studies showed that indoor radon concentration was found to increase with the gamma dose rate (Fujimoto, 1998) and that the indoor radon concentration has a coarse correlation with the gamma dose rate (Iimoto et al., 2001;  Kurnaz et al., 2011; Pilkyte & Buktus, 2005; Sundal & Strand, 2004).

### Autocorrelation function for gamma radiation dose rates

The autocorrelation function (ACF) indicates how spatially neighbouring values depend on each other or temporal values depend on preceding ones. When data have a trend, the autocorrelations for small lags tend to be large and positive because observations nearby in time are also nearby in size. So, the ACF of trended time series tends to have positive values that slowly decrease as the lags increase. When data are seasonal, the autocorrelations will be more significant for the seasonal lags (at multiples of the seasonal frequency) than for other lags. When data are both trended and seasonal, it is possible to see a combination of these effects where the slow decrease in the ACF, as the lags increase, is due to the trend, while the “scalloped” shape is due to the seasonality. Many time series also exhibit cycle behaviour which is different from seasonal behaviour. Seasonality is always of a fixed and known period, while a cycle occurs when the data exhibit rises and falls that are not of a fixed frequency (Hyndman & Athanasopoulos, 2021). The partial autocorrelation (PACF) at lag *k* is the correlation that results after removing the effect of any correlations due to the terms at shorter lags. Both autocorrelation functions (ACFs) and partial autocorrelation functions (PACFs) are usually used in the time series analysis to identify the patterns in the data, including seasonal cycles and trends.

Time series that show no autocorrelation is called white noise. For white noise series, each autocorrelation should be close to zero but not precisely equal to zero as there is some random variation. For a white noise series, approximately 95% of the spikes in the ACF should lie within ± 2/√*T*, where *T* is the length of the time series. If one or more large spikes are outside these bounds, or if substantially more than 5% of spikes are outside these bounds, then the series is probably not white noise (Hyndman & Athanasopoulos, 2021).

The autocorrelation function (ACF) and partial autocorrelation function (PACF) were calculated for each data set of the considered series. First, it was necessary to test the data for stationarity, assuring that the mean and the variance of each data set are not functions of time but rather are constants (or approximately constants). Two statistical tests were used to verify the stationarity of the considered series: the augmented Dickey–Fuller (ADF) and the Kwiatkowski–Phillips–Schmidt–Shin (KPSS) tests. Both tests verified the stationary of the series with a *p-value* smaller than 0.01.

**Fig. 8:**
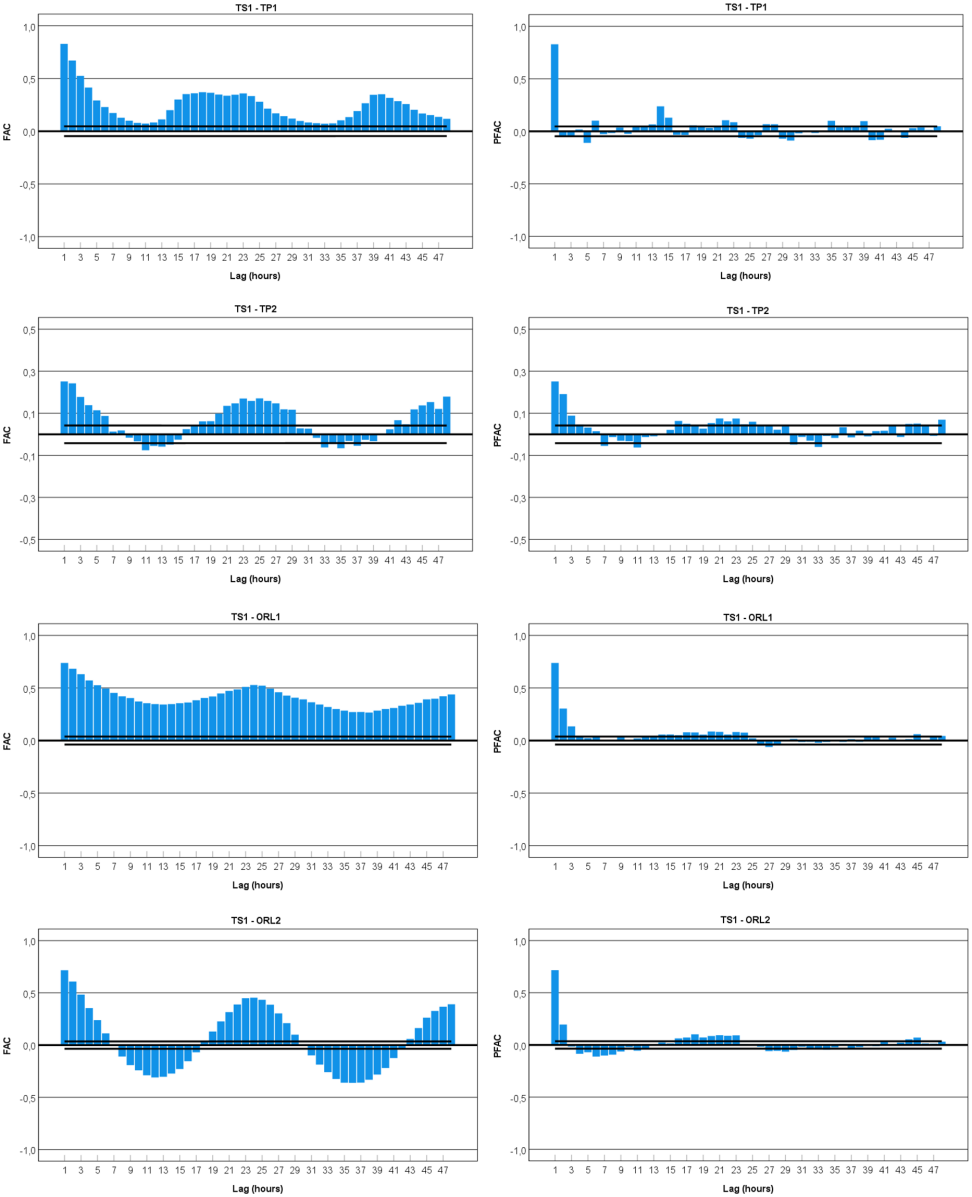
Autocorrelation function (ACF) and partial autocorrelation function (PACF) plots for time series of hourly gamma dose rates (TS1-TP1, TP2, ORL1, ORL2)

**Fig. 9:**
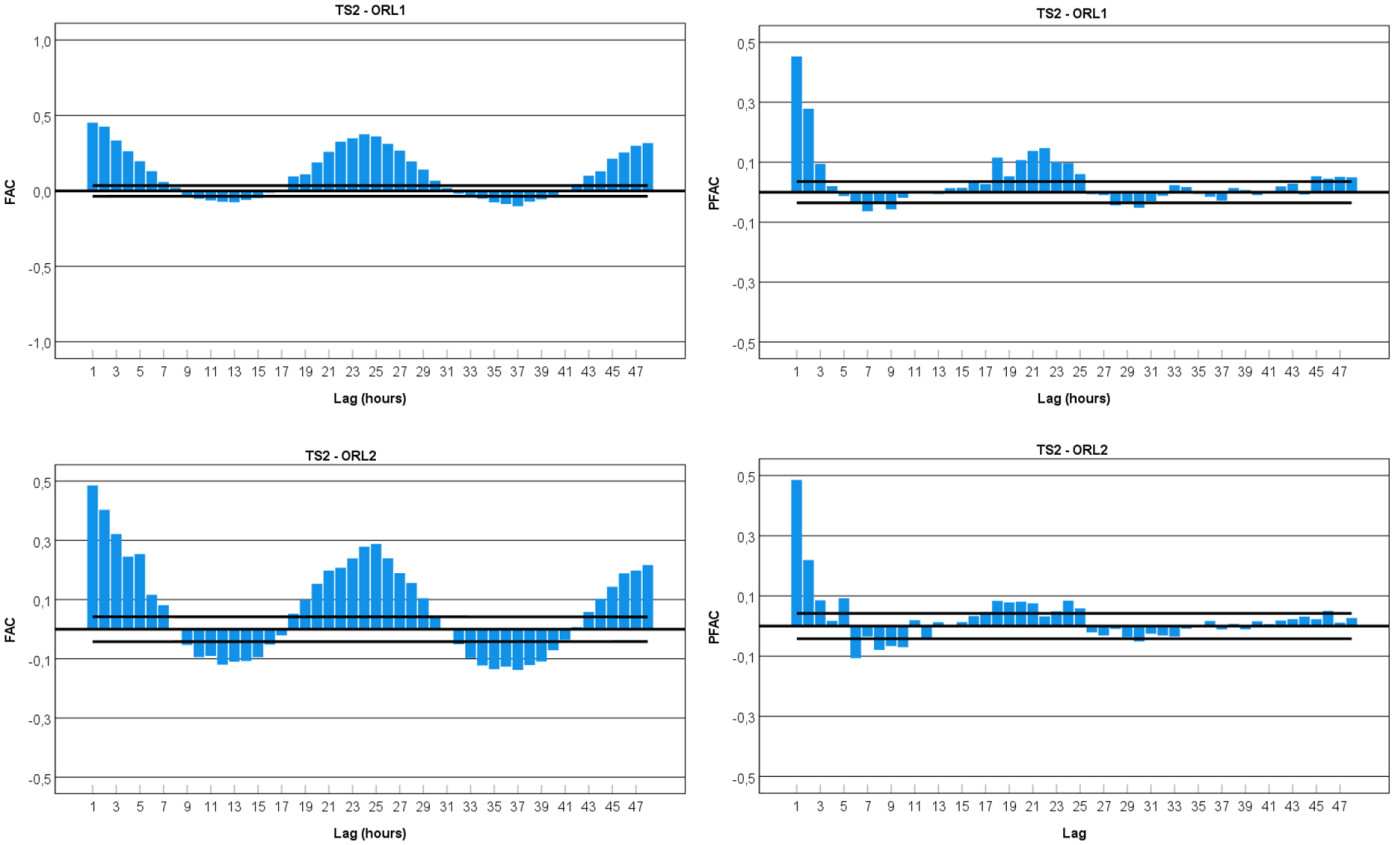
Autocorrelation function (ACF) and partial autocorrelation function (PACF) plots for time series of hourly gamma dose rates (TS2-ORL1, ORL2)

An analysis was made for the ACF and PACF to establish the potential adjustment of the hourly gamma dose rates to an ARIMA model (auto-regressive integrated moving average) or a SARIMA model (seasonal autoregressive integrated moving average). Seasonal decomposition was applied to the data set before proceeding with the analysis. The plots for the ACF and PACF of the considered time series are presented in Figs. 8 and 9 for TS1 and TS2, respectively.

In general, all ACF plots present smooth peaks, typically lasting a few hours. The analysis of the correlograms TS1-TP1 and TS1-TP2 highlights the presence of seasonal cycles. The wave for TS1-TP1 correlogram shows spikes in the seasonal lags 1 (0.829), 18 (0.370), and 40 (0.351). In this case, the pattern is a positive wave with some decaying effect. For TS1-TP2 correlogram, it is possible to observe sine spikes in the lags of 1 (0.252), 25 (0.171), and 48 (0.179). The pattern is a sine wave with some decaying effect. This effect significantly supports the evidence of a cycle effect in the data sets from the thermal pool of TS1.

The correlogram of the data sets from TS1-ORL1 and TS1-ORL2 shows a 24-h periodicity with some decay effect over time. For the pattern, it is possible to observe a smooth, positive wave for the winter/spring period (TS1-ORL1) and a sine wave for spring/summer data sets (TS1-ORL2). The first case presents spikes in the seasonal lags 1 (0.738), 24 (0.528), and 48 (0.439) and the second case 1 (0.741), 24 (0.542), and 48 (0.435). This effect significantly supports the evidence of periodicity in the data registered in the ORL of TS1.

The correlogram plot of the TS2-ORL1 data set depicts a sine wave and shows spikes in the seasonal lags 1 (0.267), 24 (0.176), and 48 (0.164). This effect significantly supports the evidence of a 24-h periodicity in the data set from the ORL of TS2.

For the data set from the TS2-ORL2, the lagged autocorrelations are all close to zero, and therefore this time series is probably white noise. The white noise can be confirmed by calculating the limits ± 2/√*T*, where *T* is the length of the time series (it is expected that 95% of the spikes in the ACF lie within these bounds) (Shumway & Stoffer, 2017). For this data set (TS2-ORL2), *T* = 2254 and, therefore, the error bounds are ± 0.042. All autocorrelation coefficients lie within these limits, confirming that the data are white noise.

The study of the correlograms may suggest the use of seasonal ARIMA (*p*,*d*,*q*) (*P*,*D*,*Q*)_*s*_ models (SARIMA) (*seasonal*, *autoregressive*, *integrated*, *moving average*). The *autoregressive terms* (AR) (*p* and seasonal *P*) is the number of terms in the model that describe the dependency among successive observations (lags of the stationary series); the *integrated terms* (*d* and seasonal *D*) are the terms needed to make a nonstationary time series stationary (differencing that must be done to stationary series) and the *moving average terms* (MA) (*q* and seasonal *Q*) are the number of terms that describe the persistence of a random shock from one observation to the next (lags of the forecast errors), and *s* is the length of the seasonal cycle or period.

The next step is to estimate the parameters of the model (*p*,*d*,*q* and *P*,*D*,*Q*). If the time series is stationary, there is no need for trend differentiating; therefore, *d* is zero. On the other hand, usually, the seasonal part of an ARIMA model has the same structure as the non-seasonal part: it may have an AR factor (*p*), an MA (*q*) factor, and/or an order of differencing (*D*). These three parameters will vary in the present case. An analysis of the time series plots and ACF and PACF plots is made after each variation, as ARIMA modelling is usually an exploratory procedure. If the data are poorly fit by a model, the identification process is revisited until a satisfactory model is found (Shumway & Stoffer, 2017).

The final step is to select the best parameters of SARIMA models by minimizing the forecast measure error RMSE (root mean square error), MAE (mean absolute error produced by the actual forecast), and MASE (mean absolute scaled error—a measure of the forecast accuracy). In addition, two criteria were implicit in the methodology to select the best model for each data set. The first criterion, the Akai information criterion (AIC), is an estimator of prediction error and the relative quality of statistical models for a given set of data. Given a collection of models for the data, AIC estimates the quality of each model relative to each of the other models. Thus, AIC provides a means for model selection. The second criterion, the Schwartz–Bayes criterion (SBIC), is a criterion for model selection among a finite set of models; the lowest SBIC is preferred. It is based, in part, on the likelihood function, and it is closely related to the AIC (Shumway & Stoffer, 2017).

**Fig. 10:**
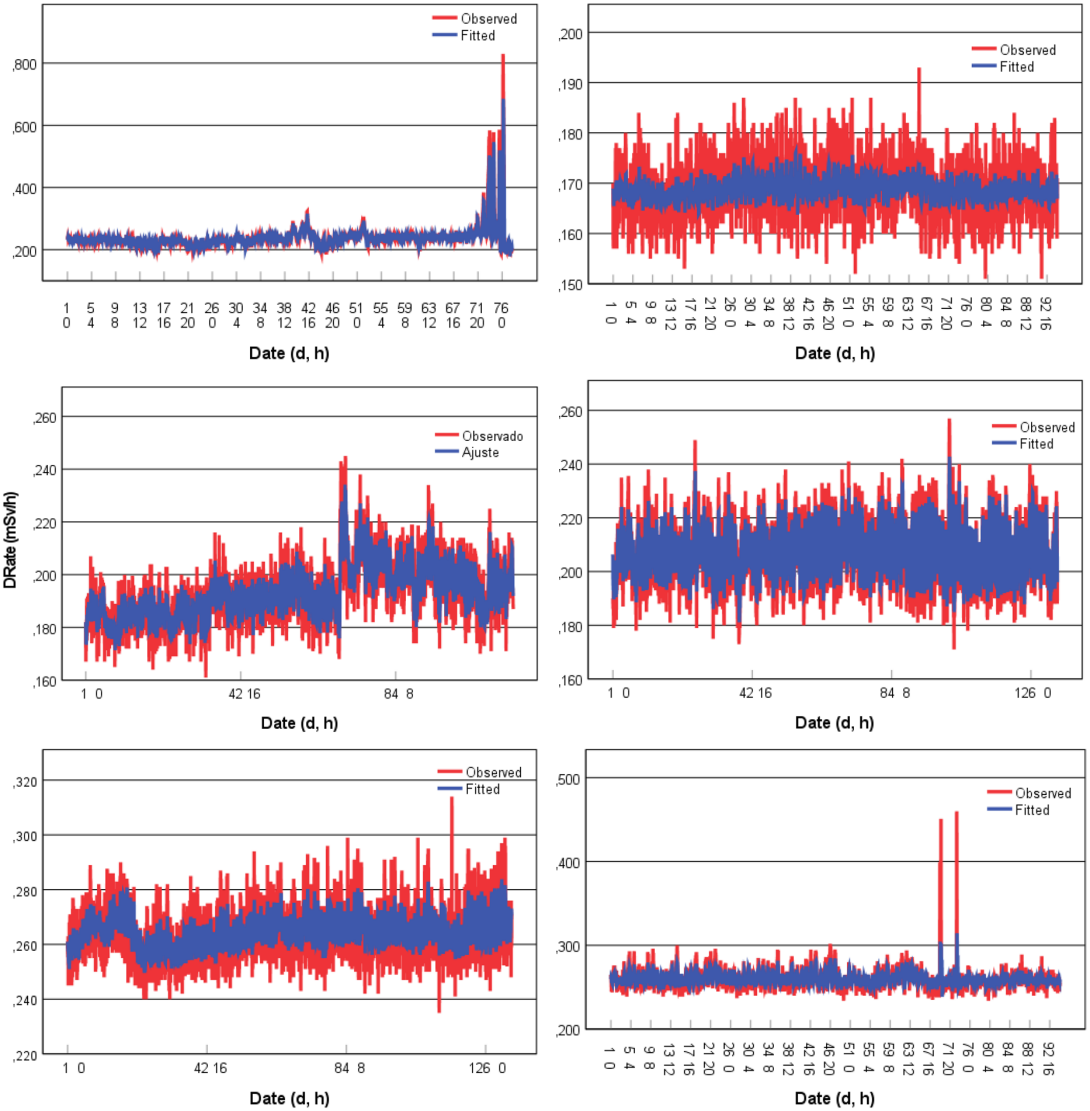
Time-series (ARIMA model) fitted to the gamma dose rates (µSv/h) at TS1 (TP1, TP2, ORL1, and ORL2) and TS2 (ORL1 and ORL2)

The most appropriate models were obtained and are presented in Table 5. For two data sets (TS1-ORL1 and TS2-ORL1), it was necessary to differentiate the time series to make the series stationary as fitted by the respective model. The solutions for the fitted models are plotted in Fig. 10.

**Table 5:** ARIMA models parameters adjusted to the data set

Data set	Model	RMSE	MAE	MASE	BIC
TS1-TP1	ARIMA (1,0,13) (1,0,0)	0.021	0.009	3.584	−7.744
TS1-TP2	ARIMA (3,0,0) (1,0,1)	0.005	0.004	2.527	−10.434
TS1-ORL1	ARIMA (0,1,5) (1,0,1)	0.007	0.006	2.975	−9.836
TS1-ORL2	ARIMA (1,0,1) (1,0,1)	0.011	0.007	2.539	−9.078
TS2-ORL1	ARIMA (0,1,9) (1,0,1)	0.007	0.006	2.219	−9.788
TS2-ORL2	ARIMA (0,0,7) (1,0,1)	0.008	2.916	0.006	−9.749

**Fig. 11:**
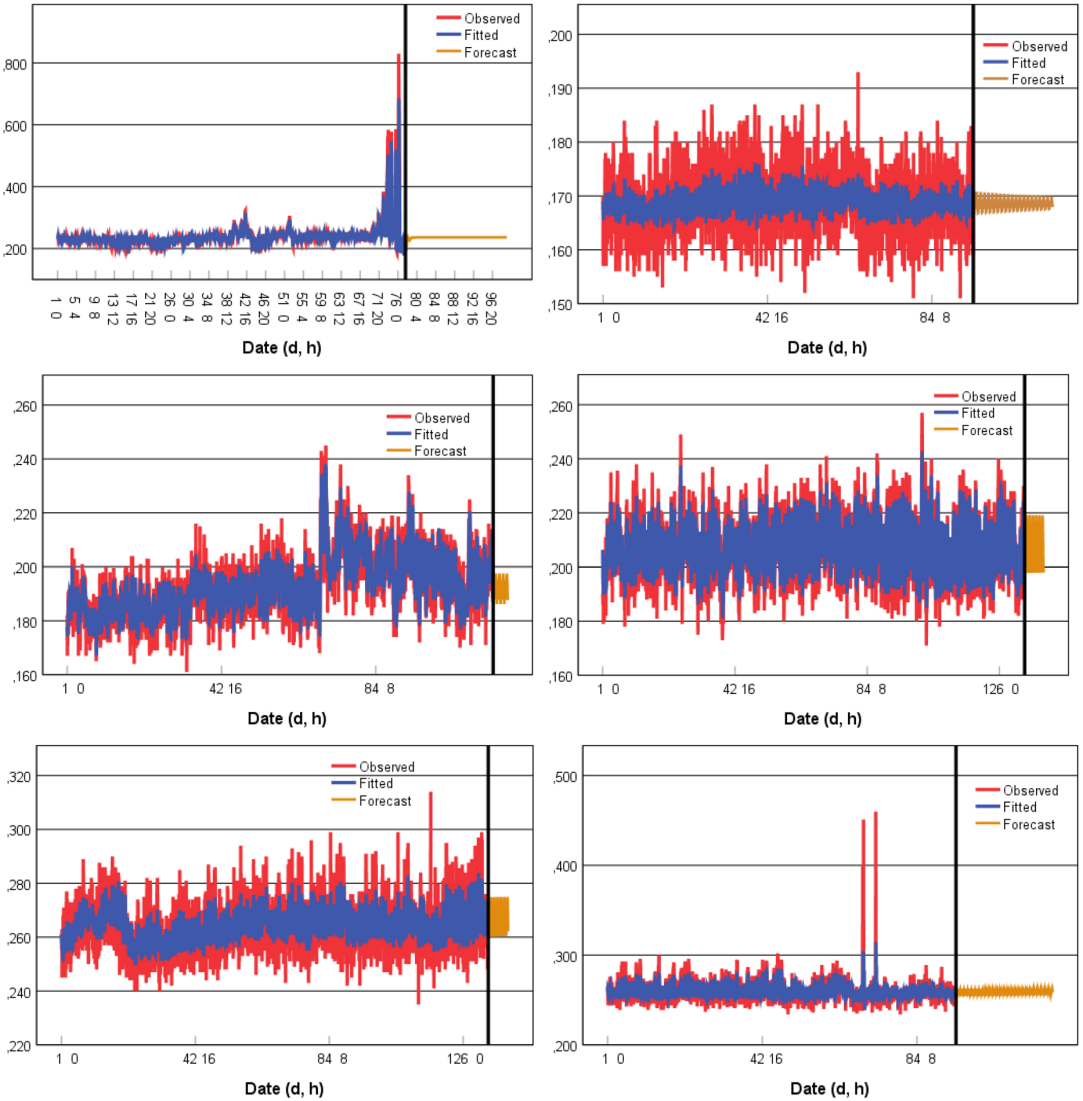
Time-series (ARIMA model) prediction of gamma dose rates (µSv/h) at TS1 (TP1, TP2, ORL1, and ORL2) and TS2 (ORL1 and ORL2)

For each case, the ARIMA prediction of hourly values for the following 7 to 10 days (168–240 h) is presented in Fig. 11. For the horizontal axis, the first line represents the day, while the second line represents the hour.

ARIMA modelling is a trial-and-error process in which successfully more complex models are fit until the residuals show no further structure (significant autocorrelations). Sometimes a time series cannot be identified as an ARIMA model. This highlights the fact why ARIMA modelling should be used as an exploratory procedure (Shumway & Stoffer, 2017). The application of ARIMA modelling in this study to find a suitable model for each case was intended by no means to be exhaustive.

For the forecast, predicting future observations from a known time series (prediction beyond the data) should be approached with caution. The farther the prediction beyond the actual data, the less reliable the prediction is. And only a small percentage of the actual data points can be predicted before the forecast turns into a straight line. This can be observed in the first case (TS1-TP1), where after a few oscillations, the model draws a straight line. The last model should not be taken into consideration as it was interpreted as white noise. The correlation coefficient between the observed data and the fitted values showed a strong correlation for TS1-TP1, TS1-ORL1, TS1-ORL2, and TS2-ORL1: 0.95, 0.88, 0.71 and 0.70, respectively.

## Conclusions

The current results support the conclusions of previous studies and provide data on occupational exposure to radon, obtained continuously and over 1 year, for three Portuguese thermal spas. The results reveal that in almost all locations of the TS1 and TS3, radon concentration levels in the indoor air are much higher than the reference level provided in the Decree-Law No. 108/2018 (300 Bq/m^3^). For example, in the ORL from TS1, radon levels are 100 times higher than in ORL from TS2. For many of these locations (9 in 20), the estimated dose is higher than 6 mSv/year, and, following the Euratom Directive 2013/59, these exposures shall be managed as a planned exposure situation. Given this scenario, mitigation actions shall be taken to reduce the concentration of radon in the workplace and keep it below the reference level. Such actions may include changes in the workplace conditions or in the conditions of the building that may lead to changes in the radon exposure.

With the exception of the registered overflow of 3.366, in all locations, the gamma dose rates were below 1 μSv/h, so the contribution to the annual effective dose is negligible. However, the variation of gamma dose rates showed some interesting disturbances, indicating a relationship with the potential accumulation of radon progeny in some particular situations, such as during some periods when the facility was closed.

The time series analysis allowed to fit some models to the gamma dose rate variation, and although the obtained models cannot predict the exact gamma dose rates, they can give information that helps to establish strategies for proper planning or be used as a supplemental tool for planning and decision-making in occupational exposure. Despite the significant abnormal values observed in TS1-TP1, the correlation between the observed and fitted data is 0.95. For the other cases, the correlation ranges between 0.70 and 0.88.

## Data Availability

All data generated or analyzed during this study are included in this published article.

## References

[CR1] Alali AE, Al-Shboul KF, Yaseen QB, Alaroud A (2019). Assessment of radon concentrations and exposure doses in dwellings surrounding a high capacity gas turbine power station using passive measurements and dispersion modelling. J of Environ Radioact.

[CR2] Alghamdi AS, Aleissa KA, Al-Hamarneh IF (2019). Gamma radiation and indoor radon concentrations in the western and Southwestern regions of Saudi Arabia. Heliyon.

[CR3] Allab, M. (2019). Preliminary study of effect of environmental parameter variations on indoor radon concentrations in Mediterranean climate. *International Journal of Low Radiation, 11*(2).

[CR4] Berens, A. S., Diem, J., Stauber, C., Dai, D., Foster, S., & Rothenberg, R. (2017). The use of gamma-survey measurements to better understand radon potential in urban areas. *Science of the Total Environment,* 607–608, 888–899. 10.1016/j.scitotenv.2017.07.022 10.1016/j.scitotenv.2017.07.022 PMC5613979 28711851

[CR5] Box GEP, Jenkins G (1970). Time series analysis: Forecasting and control.

[CR6] Box GEP, Jenkins G, Reinsel G (2008). Time series analysis.

[CR7] Box GEP, Tiao GC (1975). Intervention analysis with applications to economic and environmental problems. JASA.

[CR8] Burnett, J. L., Croudace, I. W., & Warwick, P. E. (2010). Short-lived variations in the background gamma-radiation dose. *Journal of Radiological Protection, 30,* 525. 10.1088/0952-4746/30/3/007 10.1088/0952-4746/30/3/007 20826890

[CR9] Dinis, M. L., & Silva, A. S. (2018). Radiological characterization of the occupational exposure in hydrotherapy spa treatments. In P. Arezes, J. S. Baptista, M. Barroso, P. Carneiro, P. Cordeiro, N. Costa, R. Melo, A. S. Miguel, & G. Perestrelo (Eds.), *Occupational safety and hygiene*. 10.1201/9781351008884

[CR10] Domingos, F., Cinelli, G., Neves, L., Pereira, A., Braga, R., Bossew, P., & Tollefsen, T. (2020). Validation of a database of mean uranium, thorium and potassium concentrations in rock samples of Portuguese geological units, generated of literature data. *Journal of Environmental Radioactivity, 222*. 10.1016/j.jenvrad.2020.106338 10.1016/j.jenvrad.2020.106338 32836144

[CR11] Duarte, D. C. R. (2013). Radioactive studies in Porto Urban Area: Gamma radiation of the Paranhos Sector. Master dissertation in Geotechnical Engineering and Geoenvironment, ISEP - Instituto Superior de Engenharia do Porto.

[CR12] Fujimoto, K. (1998). Correlation between indoor radon concentration and dose rate in air from terrestrial gamma radiation in Japan. *Health Physics, 75*(3), 291–296. 10.1097/00004032-199809000-00008 9721838

[CR13] Hyndman, R. J., & Athanasopoulos, G. (2021). Forecasting: Principles and practice, 3rd edition. OTexts: Melbourne, Australia. https://otexts.com/fpp3/. Accessed 21 August 2021.

[CR14] IAEA. (2014). Safety Standards Radiation for protecting people and the environment. Radiation Protection and Safety of Radiation Sources: International Basic Safety Standards Interim edition NGSR Part 3 (Interim), Vienna.

[CR15] ICRP. (2018). Summary of ICRP recommendations on radon. ICRP ref 4836–9756–8598. January 26, 2018. Available at: http://www.icrpaedia.org/images/f/fd/ICRPRadonSummary.pdf. Accessed 21 August 2021.

[CR16] Iimoto T, Kosako T, Sugiura N (2001). Measurements of summer radon and its progeny concentrations along with environmental gamma dose rate in Taiwan. Environ. Radioact..

[CR17] Kurnaz A, Kucukomeroglu B, Cevik U, Celebi N (2011). Radon level and gamma doses in dwellings of Trabzon, Turkey. Appl. Radiat. Isotopes.

[CR18] Marley F (1999). Investigation of atmospheric, mechanical and other pressure effects influencing the levels of radon and radon progeny in buildings. Health Physics.

[CR19] Marley F, Denman AR, Phillips PS (2000). Examination of the influence of water-heated central heating systems on the levels of radon and progeny in the workplace. Radiation Measurements.

[CR20] Nivolov J, Todorovic N, Pantic TP, Forkapic S, Mrdja D, Bikit I, Krmar M, Veskovic M (2012). Exposure to radon in the radon spa Niska Banja. Serbia. Radiat. Meas..

[CR21] Panatto, D., Ferrari, P., Lai, P., & Gallelli, G. (2006). Relevance of air conditioning for 222radon concentration in shops of the Savona Province, Italy. *Science of the Total Environment,* *33*(1–3): 25–30. 10.1016/j.scitotenv.2005.03.005 10.1016/j.scitotenv.2005.03.005 15893366

[CR22] Pilkyte L, Butkus D (2005). Influence of gamma radiation of indoor radon decay products on absorbed dose rate. J. Environ. Eng. Land. Manag..

[CR23] Pugliese M, Quarto M, Rica V (2014). Radon concentrations in air and water in the thermal spas of Ischia Island. Indoor and Built Environment.

[CR24] Robertson A, Allen J, Laney R, Curnow A (2013). The cellular and molecular carcinogenic effects of radon exposure: A review. International Journal of Molecular Sciences.

[CR25] Shumway, R. H., & Stoffer, D. S. (2017). Time series analysis and its applications, with R examples. Springer, Cham (Publisher), 4^th^ edition, pp. 562. 10.1007/978-3-319-52452-8

[CR26] Silva, A. S., Dinis, M. L., & Diogo, M. T. (2013). Occupational exposure to radon in thermal spas, Book chapter in: Occupational safety and hygiene. Eds. P. Arezes, J. S. Baptista, M. Barroso, P. Carneiro, P. Cordeiro, N. Costa, R. Melo, A. S. Miguel, G. Perestrelo, pp. 273–277. ISBN: 9781138000476, London: Taylor & Francis.

[CR27] Silva, A. S., Dinis M. L., & Fiúza, A. (2014). Research on occupational exposure to radon in Portuguese thermal spas. Book chapter in: Occupational safety and hygiene II, ed. P. Arezes, J. S. Baptista, M. Barroso, P. Carneiro, P. Cordeiro, N. Costa, R. Melo, A.S. Miguel, G. Perestrelo, pp. 323–328. ISBN: 978–1–138–00144–2, London: Taylor & Francis.

[CR28] Silva A. S., Dinis M. L., & Pereira, A. (2020). Indoor radon levels in thermal spas and the compliance with the European BSS directive: A Portuguese case study. In: Arezes P. et al. (eds). *Occupational and Environmental Safety and Health II. Studies in systems, decision and control, 277*. Springer, Cham. 10.1007/978-3-030-41486-3_18

[CR29] Silva, A. S., & Dinis M. L. (2015a). The presence of radon in thermal spas and their occupational implications – A review, Book chapter in: Occupational Safety and Hygiene III, Eds. P. Arezes, J. S. Baptista, M. Barroso, P. Carneiro, P. Cordeiro, N. Costa, R. Melo, A.S. Miguel, G. Perestrelo, pp. 353–355, ISBN 978–1–138–02765–7, London: Taylor & Francis.

[CR30] Silva, A. S., & Dinis, M. L. (2015b). Evaluation of the concentration of radon in the natural mineral water of Portuguese thermal spas, Book chapter in: Occupational safety and hygiene III, Eds. P. Arezes, J. S. Baptista, M. Barroso, P. Carneiro, P. Cordeiro, N. Costa, R. Melo, A.S. Miguel, G. Perestrelo, pp. 353–355, ISBN 978–1–138–02765–7, London: Taylor & Francis.

[CR31] Silva, A. S., Dinis, M. L., & Pereira, A. J. S. C. (2016). Assessment of indoor radon levels in Portuguese thermal spas. *Radioprotection*, pp. 249–254. 10.1051/radiopro/2016077

[CR32] Silva, A. S., & Dinis, M. L. (2016). Measurements of indoor radon and total gamma dose rate in Portuguese thermal spas, Book chapter in: Occupational safety and hygiene IV, Eds. P. Arezes, J. S. Baptista, M. Barroso, P. Carneiro, P. Cordeiro, N. Costa, R. Melo, A. S. Miguel, G. Perestrelo, pp. 485–489, ISBN 9781138029422, London: Taylor & Francis. 10.1201/b21172-93

[CR33] Silva, A. S., & Dinis, M. L. (2017a). Indoor radon in dwellings: an increment to the occupational exposure in Portuguese thermal spas. Occupational Safety and Hygiene V.

[CR34] Silva, A. S., & Dinis, M. L. (2017b). Variability of indoor radon level accumulation: A study in Portuguese thermal spas. *RAD Conference Proceedings, 2,* 41–148.

[CR35] Silva, A. S., & Dinis, M. L. (2018a). Main mitigation measures – occupational exposure to radon in thermal spas. Occupational Safety and Hygiene VI, pp. 425–429, London: Taylor & Francis, ISBN 978–1–138–54203–7.

[CR36] Silva, A. S., & Dinis, M. L. (2018b). A influência das condições geológicas na concentração de radão no ar interior dos estabelecimentos termais. Book chapter in: Occupational safety and hygiene, Eds. P. Arezes, J. S. Baptista, M. Barroso, P. Carneiro, P. Cordeiro, N. Costa, R. Melo, A. S. Miguel, G. Perestrelo, pp.117–119. ISBN: 978–989–98203–8–8.

[CR37] Silva, A. S., & Dinis, M. L. (2018c). Radiological characterization of Portuguese natural mineral water. *RAD 2018c Conference Proceedings, 3*, 106–110. ISSN 2466–4626 (online). 10.21175/RadProc.2018c.23

[CR38] Steck, D. J., Sun, K., & Field, RW. (2019). Spatial and temporal variations of indoor airborne radon decay product dose rate and surface-deposited radon decay products in homes. *Health Physics, 116*(5), 582–589. 10.1097/HP.0000000000000970 10.1097/HP.0000000000000970 PMC7141775 30747753

[CR39] Sun, K., Field, R. W., & Steck, D. J. (2010). Room model based Monte Carlo simulation study of the relationship between the airborne dose rate and the surface-deposited radon progeny. *Health Physics, 98*(1), 29–36. 10.1097/HP.0b013e3181b8cf92 10.1097/HP.0b013e3181b8cf92 19959948

[CR40] Sundal, A. V., & Strand, T. (2004). Indoor gamma radiation and radon concentrations in Norwegian carbonate area. *Journal of Environmental Radioactivity, 77*, 175–189. 10.1016/j.jenvrad.2004.03.007 15312702

[CR41] UNSCEAR. (2017). Publication Report Sources, Effects and Risks of Ionizing Radiation, United Nations, New York.

[CR42] UNSCEAR. (2000). Publication E.00.IX.3. Sources and effects of 91 ionizing radiation, United Nations, New York.

[CR43] Vukotic P, Antovic N, Djurovic A, Zekic R, Svrkota N, Andjelic T, Svrkota R, Mrdak N, Bjelica N, Djurovic T, Dlabac A, Bogicevic M (2019). Radon survey in Montenegro – A base to set national radon reference and “urgent action” level. J of Environ Radioact.

[CR44] WHO. (2009). *Handbook on indoor radon: A public health perspective, 93*. H. Zeeb, F. Shannoun, Eds. 23762967

[CR45] Xie, D., Maili, L., & Kearfott, K. J. (2015). Influence of environmental factors on indoor radon concentration levels in the basement and ground floor of a building – A case study. *Radiation Measurements, 82*, 52–58. ISSN 1350–4487. 10.1016/j.radmeas.2015.08.008

